# Linear Reciprocating Tribometer for In Situ Neutron Reflectometry of Soft Matter

**DOI:** 10.1007/s11249-025-02049-1

**Published:** 2025-07-29

**Authors:** Kathryn E. Shaffer, Brendan Louie Bagorio, Ahmed Al Kindi, Julia J. Ong, Andrew R. Rhode, Erik B. Watkins, Rebecca J. L. Welbourn, Roger Pynn, Juan Manuel Urueña, Angela A. Pitenis

**Affiliations:** 1 Materials Department, University of California, Santa Barbara, Santa Barbara, CA 93106, USA; 2 Department of Mechanical Engineering, University of California, Santa Barbara, Santa Barbara, CA 93106, USA; 3 Oak Ridge National Laboratory, Oak Ridge, TN 37831, USA; 4 Materials Research Laboratory, University of California, Santa Barbara, Santa Barbara, CA 93106, USA; 5 Department of Physics, Indiana University Bloomington, Bloomington, IN 47405, USA; 6 NSF BioPACIFIC Materials Innovation Platform, University of California, Santa Barbara, Santa Barbara, CA 93106, USA

**Keywords:** Tribometer design, Neutron reflectometry, Soft matter

## Abstract

Neutron reflectometry is a technique for measuring structure near planar interfaces that has been previously used to non-destructively characterize the polymer density of hydrated, dilute, and soft materials. Previous investigations have conducted neutron reflectometry measurements of liquids, gels, emulsion, and polymer solutions at rest, in compression, and subject to shear stress. However, correlating structure with tribological properties of soft materials presents significant experimental challenges for prior instruments due to wall slip, sample thickness, and structural heterogeneity (e.g., depth-wise gradients). A linear reciprocating tribometer offers several advantages for in situ neutron reflectometry studies, including uniform velocity profiles, constant shear stress over large regions of interest, and independent control of normal force and sliding velocity during measurements. This work outlines basic considerations for the design of a custom linear reciprocating tribometer that operates in a neutron beamline and includes commissioning measurements. The tribometer is designed to compress soft and hydrated materials against linearly reciprocating silicon disks. The three key design considerations for this tribometer are (1) safety, (2) neutron transmission, and (3) sample positioning. This instrument design will enable in situ studies of soft matter and illuminate the role of interfacial structure on tribological phenomena.

## Graphical Abstract

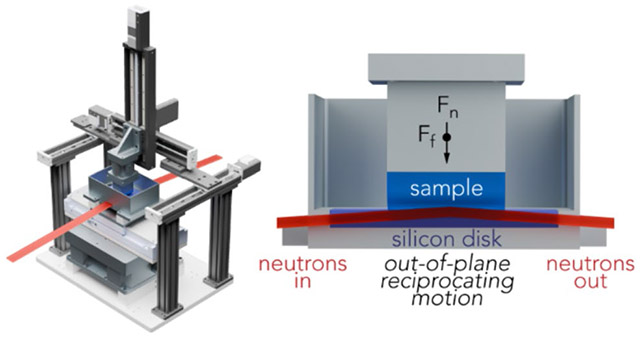

## 1 Introduction

Neutron reflectometry is an elastic scattering technique that has been used to characterize the interfacial structure of soft materials, from lipids to gels [1-10]. This technique is used to measure the depth resolved composition (e.g., polymer density) of samples averaged over a surface area on the order of square centimeters with nanoscale depth resolution, allowing detailed investigations of structure as a function of depth up to hundreds of nanometers into a sample. Neutrons are particularly well suited for investigating soft matter; a neutron beam is largely nondestructive compared to other probes (e.g., X-rays, electrons), which preserves the structural integrity of soft materials throughout experiments. Additionally, the neutron scattering signal does not scale in a simple way with atomic number as it does with X-rays; therefore, materials containing constituents with similar atomic numbers (e.g., H, C, N, O) or different isotopes (e.g., ^1^H vs. ^2^H) can have resolvable, unique signals in neutron reflectometry [11, 12].

Sample environments, or in situ devices, have been widely used to investigate both stationary and dynamic interfaces of soft materials with neutron reflectometry [13]. Among these are passive load chambers which have measured thermally fluctuating soft materials (e.g., hydrogels) at rest and in static compression [14, 15]. Flexible inflatable membranes have been used to investigate the structure of thin soft matter films and polymers under confinement [16]. Flow cells have been used to impose shear stress upon polymer solutions [17, 18], while Langmuir troughs have been commonly used to compress and deform monolayers and multilayers suspended on fluids [19]. Rheometers have been extensively used to investigate the structure of fluids, polymers, and suspensions under shear [20-23, 42] and the emergence of new tribolayers [24]. Recently, an innovative device in the style of a rolling pin was used to characterize the structure of solid–liquid interfaces under shear [25].

Linear reciprocating friction-measuring devices in a neutron reflectometer, to the best of the authors’ knowledge, are absent from the literature. A linear reciprocating tribometer designed for neutron reflectometry enables surface structure investigations during measurements of friction, deformation, and wear of surfaces sliding in relative motion. Linear reciprocating motion achieves uniform velocity profiles over large regions of interest (≈1500 mm^2^) averaged in neutron reflectivity data. Sliding velocity and normal force are independently controlled during friction measurements. This instrument could be used to investigate samples that have been investigated with rheometers, flow cells, Langmuir troughs, and rolling pin style tribometers (e.g., liquids, gels, emulsions, and solutions). In addition to these materials, this instrument could be used to examine samples that exhibit wall slip (e.g., lubricious interfaces) as well as samples that are thin, thick, and/or composed of depth-wise gradients. Measuring forces across surfaces in relative motion presents unique experimental challenges when coupled with neutron reflectivity. This paper outlines considerations for designing a linearly reciprocating tribometer for in situ neutron reflectivity studies, enabling future insights into the structure of dynamic soft matter interfaces.

## 2 Neutron Beamline Considerations

### 2.1 Safety

Special considerations must be taken in operating a tribometer within a neutron beamline. Most importantly, neutrons are a radiation hazard. Therefore, the operation of the tribometer must be done remotely to eliminate human exposure to the radiation fields. Due to this constraint, additional positioning stages beyond the tribometer motion are required to maneuver the sliding interface into the immovable neutron beam.

Neutron activation half-life of materials within the beamline determines how long they are considered hazardous following irradiation. For this reason, the tribometer is composed of materials with irradiated half-lives on the order of minutes, such as aluminum [26, 27], whenever possible.

### 2.2 Horizontal Beamline Configuration

Linearly reciprocating tribometers, particularly those used to investigate hydrated or submerged samples, are typically designed such that sliding interfaces are perpendicular to gravity. This operation is best suited for a horizontally configured liquids reflectometer. The tribometer described herein has thus been specifically designed for use with the Oak Ridge National Laboratory (ORNL) liquids reflectometer (LIQREF) at the Spallation Neutron Source (SNS).

**Fig. 1:**
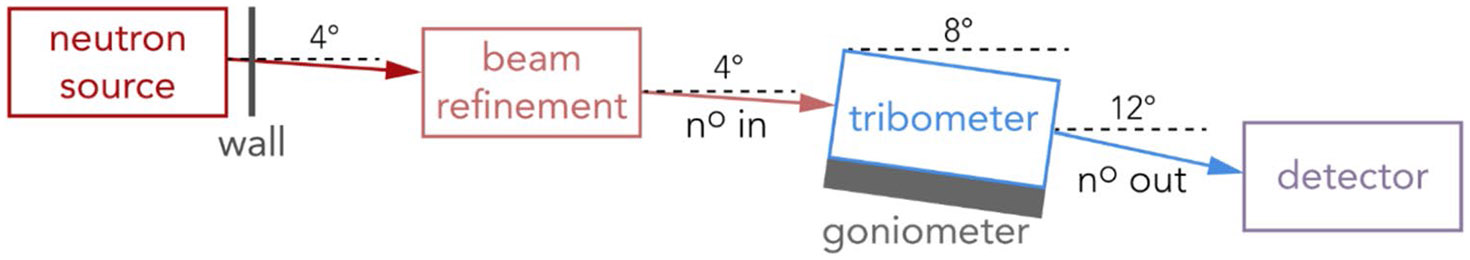
Schematic of the neutron beam path at the Oak Ridge National Laboratory liquids reflectometer interacting with the tribometer sample environment

The LIQREF neutron beam path is schematically depicted in Fig. 1. Neutrons are produced by a pulsed spallation source, cooled in a liquid hydrogen moderator, and transported to the instrument using neutron guides. The neutron wavelength distribution is defined using choppers and frame overlap mirrors, and the beam dimensions are defined using a pair of absorbing slits. The incident beam propagates at an angle of 4° below the horizontal and enters the tribometer through the side of a single-crystal silicon disk whose upper polished surface translates relative to the sample material. The neutron beam is then projected on the silicon upper surface in an approximately 20 mm wide and 40 mm long footprint.

Specular reflectivity is a specific type of elastic scattering, whereby the angle of incidence of neutrons on the sliding interface ($n^{o}$ in) matches the angle of reflection of neutrons from the interface ($n^{o}$ out). The neutron reflectivity is calculated as the reflected intensity divided by the incident intensity [28].

Neutron reflectivity is measured as a function of the momentum transfer vector, Q, perpendicular to the sliding interface. Momentum transfer depends on the angle between the neutron beam and the surface of interest, θ, and the wavelength of the neutron beam, λ (Eq. 1) [12].


(1) $$Q=\frac{4\pi \;\sin\theta }{\lambda }$$

The LIQREF operates on a pulsed spallation neutron source and uses the time-of-flight method to collect a range of neutron wavelengths from each neutron pulse. This allows Q space to be measured using both a range of λ and by manipulating the angle, θ. Precise positioning of the sample with respect to the neutron beam angle is achieved by tilting the tribometer during experiments between 0° and 4° below horizontal with respect to the neutron beam (4° to 8° with respect to gravity) using a goniometer (Fig. 1). To ensure that the sliding interface remains focused in the beam path during tilting, the sliding interface must be positioned within the goniometer’s center of rotation.

### 2.3 Materials for High Neutron Transmission to and from the Sliding Interface

When the neutron beam passes through materials to reach the sliding interface, the use of high transmission materials increases the neutron flux at the interface of interest. Silicon and aluminum have high neutron beam transmission due to low incoherent and absorption cross sections (Supplementary Table S1) [26, 29]. Because soft matter and hydrated samples typically contain large quantities of hydrogen which significantly scatters neutrons, the beam must pass through the silicon countersample to reach the sliding interface. In this tribometer design, the sample is loaded top-down onto the countersample and neutrons enter at shallow angles from below the sliding interface to avoid transmitting through the sample. To position neutrons below the sample for reflectivity measurements, the tribometer tilts from 4° to 8° below horizontal to position the beam and the detector below the sliding interface (Fig. 1). Countersamples, adapter plates, baths, and any other components located in the path of the neutron beam, excluding the sample of interest, are composed of high transmission materials to maximize flux to the sliding interface.

## 3 Tribometer Design

**Fig. 2:**
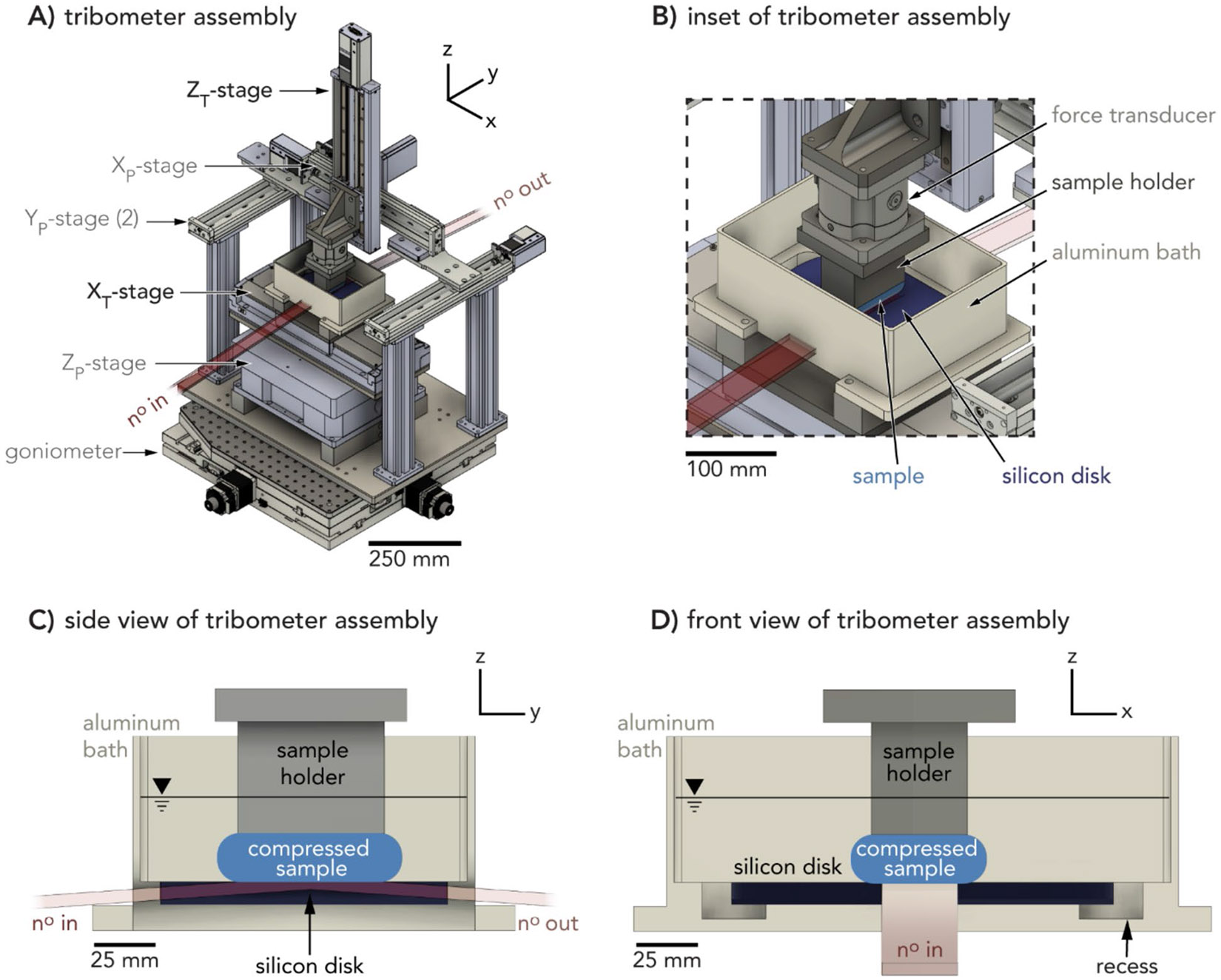
Tribometer schematic. **A** an isometric projection of the full instrument with all stages labeled. Tribometer stages necessary for compression and sliding are labeled with the axis of sliding and the letter ‘T’ subscript, while interface positioning stages are labeled with the letter ‘P’ subscript. The expected neutron beam $\left(n^{o}\right)$ path is modeled in translucent red. Direction of beam is indicated as $n^{o}$ in before neutrons interact with the sample and $n^{o}$ out after neutrons interact with the sample. **B** Inset of isometric projection in A) focused on the sliding interface. **C** Side and **D** front views of the sliding interface, shims not shown

This tribometer is designed to provide linear reciprocating motions between a stationary soft material sample compressed against a moving silicon disk countersample in a hydrated environment during neutron reflectivity measurements (Fig. 2). The sample remains fixed with respect to the neutron beamline to ensure all reflectivity data gathered are from the sample–silicon interface. Material and stage selections are optimized for neutron beam transmission. Stage and force transducer specifications are listed in Supplementary Table S2.

### 3.1 The Sliding Interface

Soft and hydrated samples, especially those without clearly distinguishable structure at the interface, generally produce smaller, less resolvable, changes in reflectivity signal. To maximize the neutron signal intensity, the neutron beam footprint must be maximized. At the LIQREF, the beam footprint is about 20 × 40 mm and the contact area between the sample and countersample must exceed this size (Fig. 2C,D). This precaution ensures that signal reaching the detector originates from the sample interface and not the surrounding region (background). The large beam footprint is fortunately amenable to soft materials, which have low elastic moduli and thus large contact areas under load. Larger contact areas also permit greater tolerance in positional uncertainty with respect to beam alignment.

The sample holder (Al 6013) attaches the sample to the tribometer and is easily removed and swapped with identical components for sample exchanges (Fig. 2). The sample holder is fastened beneath a submersible six-channel force transducer (AMTI SF3-100), which measures normal (compressive) and tangential (frictional) forces reacting across the sample during contact and sliding against the countersample (Fig. 2B).

Single crystal silicon was selected as the countersample for its high neutron transmission and its low surface roughness. Custom silicon disks (Supplementary Fig. S1, 75 mm radius, 7 mm thickness, $R_{a}<1\;\text{mm}$ on top surface), purchased from Silicon Valley Microelectronic, Inc., were designed to accommodate up to an 80 mm sliding path and a 20 mm wide neutron beam (100 mm of travel, edge-to-edge), including a free sliding regime away from reversals. The silicon disk faces on the xz plane are flattened such that when the silicon disk reciprocates with respect to the neutron beamline, the beam path length through the silicon remains the same. The thickness of the silicon disk permits incoming neutrons to enter and exit through the flattened faces away from the bottom of the disk after alignment when the last defining beam slit is positioned less than 50 mm away from the bath wall.

The sample–silicon sliding interface is situated within a solvent bath to maintain hydrated samples. The bath is machined from aluminum (Al 6061) (Fig. 2), a material selected for its high neutron transmission, low post-neutron-irradiation half-life, and low density. The walls of the bath are about 60 mm in height from the surface of the silicon disk, which supports total submersion of thick hydrated samples (about 10 mm thickness) even at a 10° tilt, which exceeds the tilt required for neutron reflectivity studies. The bath’s interior footprint (about 200 × 125 mm) accommodates an inset for the silicon disk and shallow recesses along the disk perimeter for ease of handling. The exposed interface between the aluminum and the silicon on the xz plane (Supplementary Fig. S2) is minimized with the use of aluminum shims, compressing the Si wafer into place and preventing liquid from entering the gap, which would reduce transmittance of the neutron beam. During experiments, solvent evaporation from the bath and air currents are mitigated through the use of a flexible enclosure secured to the bath rim and to the adapter plate above the force transducer.

### 3.2 Tribometer Motion Control

Two motorized positioning stages, one $Z_{T}$-stage (Zaber LRQ300AL-DE51T10A) and one $X_{T}$-stage (Physik Instrumente V-817.096211E0) (Fig. 2A), are used to control compression and sliding motions during experiments (Supplementary Fig. S3A). The ball and screw-driven $Z_{T}$-stage is attached to the force transducer which loads the sample onto the silicon disk countersample. Positioning lower on the $Z_{T}$-stage provides higher compressive forces on the sample. The $Z_{T}$-stage has < 5 μm backlash and 10 μm accuracy. The silicon disk inside the bath is mounted to a high-load, linear reciprocating, magnetic direct drive, $X_{T}$-stage. Minimum incremental motion of 0.01 μm allows for a broad range of sliding speeds.

### 3.3 Positioning the Sample in the Neutron Beam

Because the neutron beam and the center of rotation for the goniometer are in a fixed position, the sliding interface must be aligned to meet these points with 10 μm accuracy. In order to meet this requirement, the sliding interface must be able to shift positions based on neutron beam signal feedback, after the instrument cave is closed to personnel access. Combined with several adapter plates, a ball and screw-driven vertical positioning $Z_{P}$-stage (Newport IDL280-Z20) raises the top surface of the silicon disk to the targeted 330 mm height from the goniometer to the center of rotation (Fig. 2A, Supplementary Fig. S3B). The 20 mm travel range and 500 N load capacity of the stage allow the tribometer $X_{T}$-stage, bath, and silicon disk raise and lower to precisely match the center of rotation. The goniometer in LIQREF can tilt in the θ direction as well as the orthogonal χ direction, allowing for initial alignment of the silicon surface plane with the neutron beam before experiments begin.

Because the sample remains stationary with respect to the neutron beam, the sample–countersample interface must be precisely aligned in x and y such that it completely covers the neutron beam window. Three ball and screw-driven sample positioning stages (Zaber LSQ300A-E01T3A), two $Y_{P}$-stages, and one $X_{P}$-stage are mounted above the sliding interface (Fig. 2A, Supplementary Fig. S3C). These stages work together to remotely guide the tribometer $Z_{T}$-stage and mounted sample to the beam window.

## 4 Contributions to Measurement Uncertainty

### 4.1 Misalignment-Induced Force Changes due to Tilt

Soft and hydrated materials (e.g., articular cartilage, hydrogels) often exhibit low friction and thus low forces are commonly encountered in tribological measurements [30-35]. Extensive work has been put forth by others to characterize the difficulties associated with low friction measurements and to define the sensitivity of force measurements due to misalignments between the force transducer and the sliding interface [36-38]. In this tribometer design, the entire apparatus must be tilted with respect to gravity to accommodate the neutron beam path (Supplementary Fig. S4). The influence of the force of gravity on normal load measurements due to a maximum 8° tilt is considered in the following simple analysis. The change in normal load, $\Delta F_{n}$, measured by the force transducer due to tilting is a function of the combined mass of the sample and sample holder, m, and the tilt angle with respect to gravity, $\theta_{\text{tilt}}$, where g is the acceleration due to gravity (Eq. 2).


(2) $$\Delta F_{n}=mg\left(1-\cos\theta_{tilt}\right)$$

Since the maximum tilt angle of the liquids reflectometer is $\theta_{\text{tilt}}=8^{{}^{\circ}}$, the change in normal force due to this tilt angle reduces to Eq. (3):

(3) $$\Delta F_{n}\approx 0.0097mg$$

Assuming m≈0.5kg, the change in the measured normal force for this instrument configuration when tilted to $\theta_{\text{tilt}}=8^{{}^{\circ}}$ is $\Delta F_{n}\approx 0.05\;N$. Given a minimum applied normal force of $F_{n}\approx 0.5\;N$, the change in force due to the maximum tilting angle accounts for less than 10% of the applied normal force.

### 4.2 Minimum Detectable Force Measurements

**Fig. 3:**
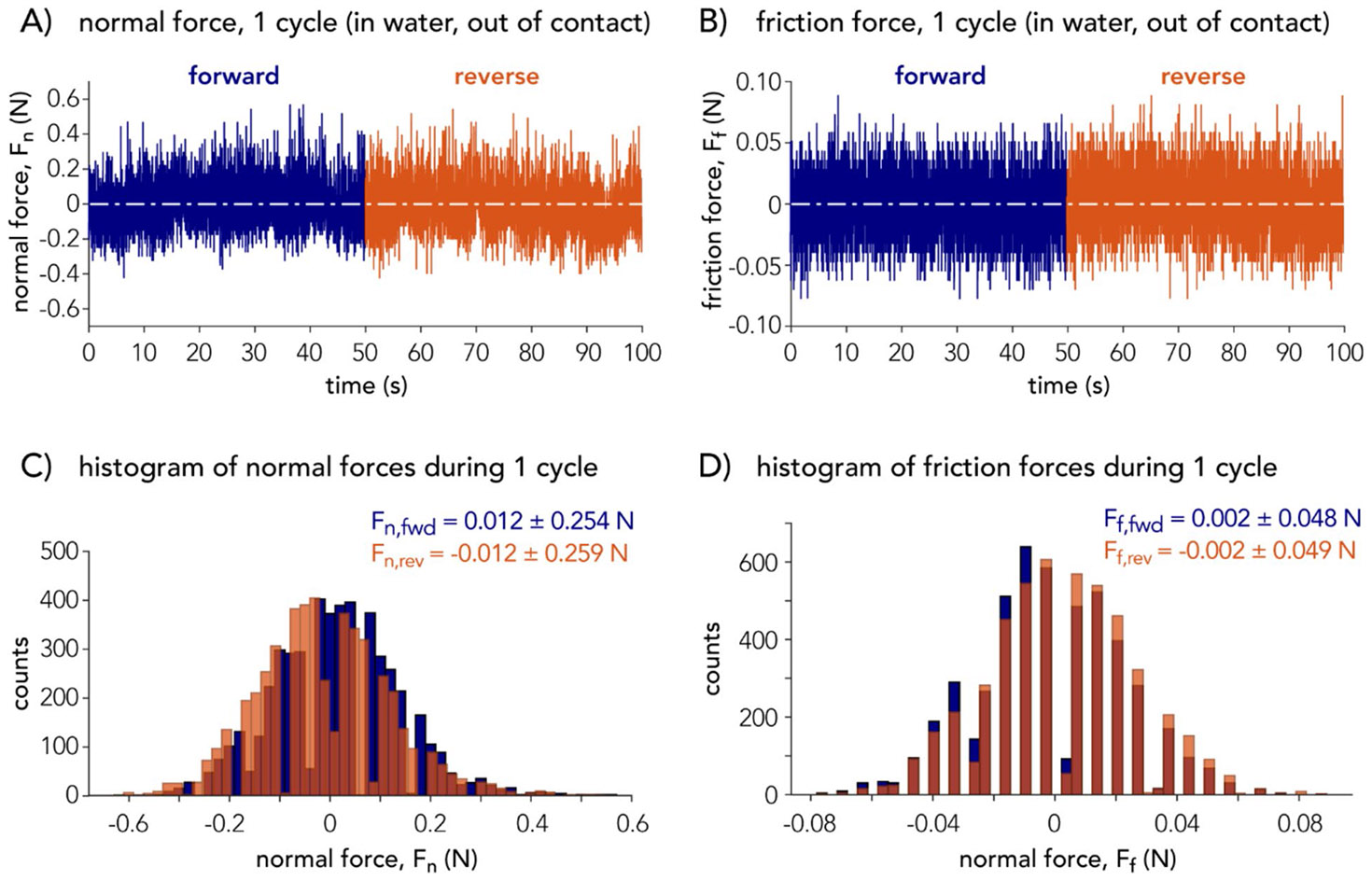
Fluctuations in (**A**, **C**) normal and (**B**, **D**) friction forces detected by the load cell due to 1 mm/s reciprocating motions with a sample holder submerged in D_2_O but out of sliding contact

To characterize the minimum detectable forces during motion in this particular tribometer, the tribometer was configured with a sample (about 60 mm in length, 40 mm wide, and 10 mm in height) mounted to the sample holder and positioned 1 mm in height away from a silicon disk. The silicon disk was secured in a water-filled aluminum bath such that the depth of the liquid from the silicon surface to the air–liquid interface was about 25 mm. The tribometer’s $X_{T}$-stage reciprocated the silicon disk beneath the suspended sample at a velocity of v=1mm∕s over a stroke length of l=50mm for one reciprocating cycle. The sensitivity and calibration constants in the normal and tangential directions of the force transducer are reported in Supplementary Table S3, and the voltage range for the operation of the load cell was 0 to 10 V. The minimum detectable experimental noise in normal and friction force measurements, respectively, were $F_{n,\text{min}}=\pm 0.261\;N$ and $F_{f,\text{min}}=\pm 0.049\;N$, based on the average of the 95% confidence interval for the forward and reverse directions (Fig. 3). This experiment was also conducted with the environmental enclosure (Supplementary Fig. S5) with negligible differences.

### 4.3 Proof-of-Principle Neutron Reflectivity Measurements of D_2_O-Silicon Interfaces

The tribometer is designed for compression and sliding experiments. Like with most sample environments, compression environments in neutron reflectometry are stationary with respect to the beam; therefore, the beam is always passing through the same materials on the way to the interface. Sliding experiments, however, require stage motion relative to the beam, adding a potential source of error to neutron data averaged over time during motion as well as potential loss of alignment to the neutron beam.

**Fig. 4:**
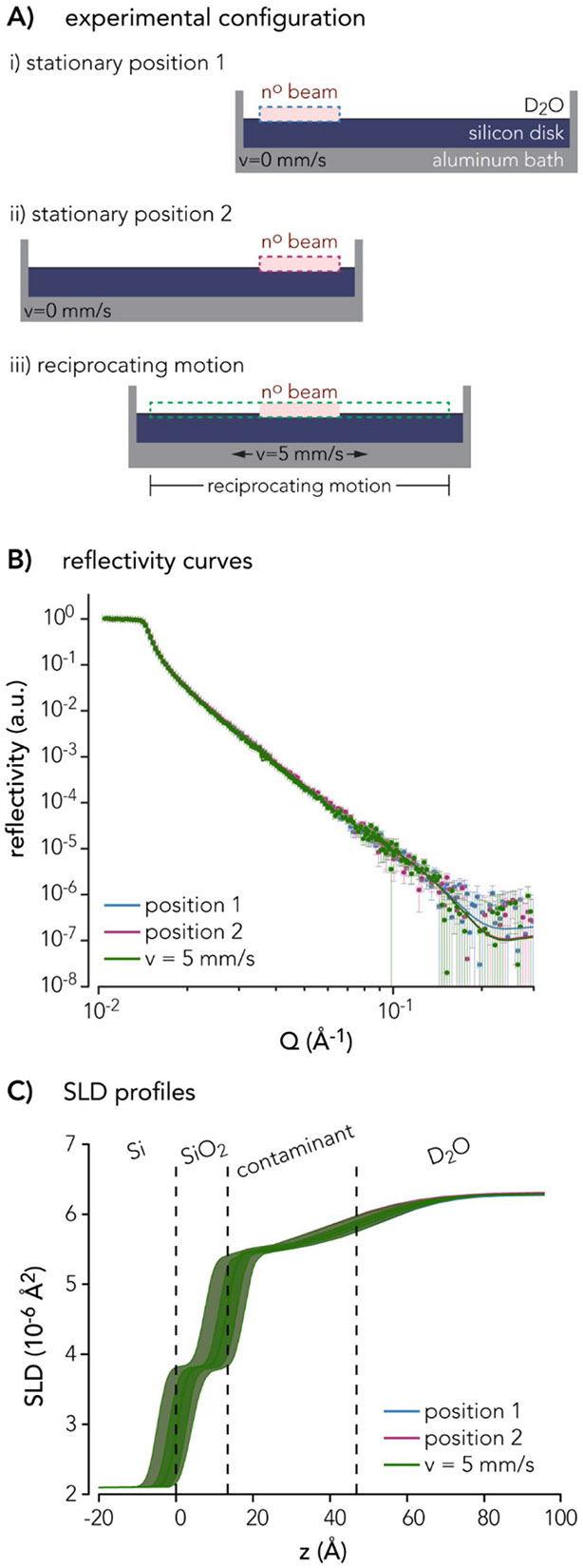
Reflectivity measurements of the D_2_O–silicon interface. **A** Schematic of the aluminum bath containing silicon disk and D_2_O with stationary neutron beam indicated by the pink box. The aluminum bath is positioned with respect to the neutron beam to probe two non-overlapping and stationary regions of interest: (i) ‘position 1,’ dashed blue region, and (ii) ‘position 2,’ dashed magenta region. The aluminum bath is depicted in (iii) during reciprocation motions at a constant sliding velocity, v=5mm∕s, while the neutron beam remains stationary, resulting in neutron reflectivity data collected over the dashed green region. **B** Log–log plot showing overlaid reflectivity curves (solid lines) fit from data points (solid circles) of position 1 (blue), position 2 (magenta), and linear reciprocating motions at v=5mm∕s (green). Error bars represent the error in reflectivity measurements. **C** Linear–linear plot showing scattering length density (SLD) profiles of the three reflectivity curves with uncertainty bands. Values for the SLD profiles can be found in Supplementary Table S4

To determine the extent to which neutron reflectivity measurements varied across the sliding path, neutron reflectivity data were collected from the D_2_O-silicon interface for three cases (Fig. 4A) after a single alignment with the LIQREF goniometer. In the first two cases, each of the two ends of the motion path (75 mm between the centers of positions 1 and 2) were observed at rest (Fig. 4Ai-ii), representing the most extreme differences in silicon alignment with respect to the neutron beam. In the third case, the D_2_O–silicon interface was probed while reciprocating at v=5mm∕s between positions 1 and 2 (Fig. 4Aiii). Representative motion profiles for the reciprocating case are shown in Supplementary Figures S6 and S7.

Reflectivity, R, was measured as a function of momentum transfer normal to the interface (Eq. 1). Here, a 2.5Å<λ<10Å Å wavelength range and three incident θ angles (0.45°, 1.2°, 3.5°) were used to measure Q up to 0.3 Å^−1^ and reflectivity down to $R\approx 5\times 10^{-7}$. The resulting overlapping neutron reflectivity curves demonstrate negligible differences in signal between the two stationary positions and during reciprocating motion (Fig. 4B), demonstrating sufficient alignment of the entire D_2_O–silicon interface with the neutron beam.

Reflectivity probes the in-plane averaged nuclear scattering length density (SLD) as a function of depth. The thickness, SLD, and roughness of a series of layers normal to the substrate were determined by minimizing the difference between the measured reflectivity and that obtained from a modeled SLD profile. Here, reflectivity data were modeled using the DREAM algorithm in REFL1D with the output parameters consistent with a D_2_O–silicon interface plus a thin, hydrogenous contaminant on the surface (Fig. 4C) [39, 40]. REFL1D is a Python package that uses the DREAM algorithm (which uses differential evolution to adapt the evolution of Markov chains) to infer the probability distribution of fit parameters and provides an error analysis that outputs uncertainty estimates for each fit parameter and a correlation plot between those parameters [41]. This proof-of-principle measurement holds promise for future tribological studies using this experimental configuration (Supplementary Fig. S8).

## 5 Limitations of the Current Tribometer Design

This tribometer is designed to measure low friction forces at relatively low contact pressures and sliding speeds. Such conditions are particularly relevant for investigations of soft and hydrated samples. Motorized stages were principally selected for smooth motions to mitigate noise in force measurements as well as for their low positional uncertainty, low backlash, and high repeatability. Stages were also selected for their ability to support the weight of several stacked components. As a result, the instrument in its current configuration cannot accommodate high-load sliding experiments $\left(F_{n}>50\;N\right)$ without exceeding factor of safety requirements. The tribometer design can be modified such that stages are substituted with more robust, load-bearing alternatives, although this may compromise the desired qualities above.

## 6 Concluding Remarks

This work introduces a new sample environment designed to achieve in situ tribological measurements on the liquids reflectometer (LIQREF) at the Oak Ridge National Laboratory (ORNL) Spallation Neutron Source (SNS) using linear reciprocation. The neutron beamline environment mandates the modification of the familiar tribometer to accommodate the following unique considerations: safety, neutron transmission, and sample positioning. We demonstrate key capabilities of the tribometer and present proof-of-principle in situ neutron reflectivity measurements of a D_2_O–silicon interface during linear reciprocating motion. We envision this instrument will enable entirely new investigations into the structure–property relationships of soft matter interfaces during dynamic loading and sliding.

## Supplementary Material

Supplementary File1

**Supplementary Information** The online version contains supplementary material available at https://doi.org/10.1007/s11249-025-02049-1.

## Data Availability

The data supporting this manuscript are freely available at Dryad, an online data repository: https://doi.org//10.5061/dryad.bg79cnpnz
