# Supplementary material for: Linear Reciprocating Tribometer for In Situ Neutron Reflectometry of Soft Matter
Source: Tribol Lett. Author manuscript; Available in PMC 2025 Sep 16. (PMC12435397; doi:10.1007/s11249-025-02049-1)
Supplement: Supplementary File1 [file NIHMS2100918-supplement-Supplementary_File1.docx]

**Supplementary Information:**

**Linear Reciprocating Tribometer for In Situ Neutron Reflectometry of Soft Matter**

Kathryn E. Shaffer^1^, Brendan Louie Bagorio^2^, Ahmed Al Kindi^2^, Julia J. Ong^1^, Andrew R. Rhode^1^, Erik B. Watkins^3^, Rebecca J. L. Welbourn^3^, Roger Pynn^4,5^, Juan Manuel Urueña^6^, and Angela A. Pitenis^1,4^

^1^ Materials Department

University of California, Santa Barbara

Santa Barbara, California, 93106, USA

^2^ Department of Mechanical Engineering

University of California, Santa Barbara

Santa Barbara, California, 93106, USA

^3^ Oak Ridge National Laboratory

Oak Ridge, Tennessee, 37831, USA

^4^ Materials Research Laboratory

University of California, Santa Barbara

Santa Barbara, California, 93106, USA

^5^ Department of Physics

Indiana University Bloomington

Bloomington, Indiana, 47405, USA

^6^ NSF BioPACIFIC Materials Innovation Platform

University of California, Santa Barbara,

Santa Barbara, California 93106, United States

**Corresponding Author**

Angela A. Pitenis

Associate Professor

Materials Department

University of California, Santa Barbara

Santa Barbara, CA 93106

email: apitenis@ucsb.edu

**Table S1:** Neutron scattering cross-sections for silicon and aluminum used for materials selection. Values from NIST Neutron Activation and Scattering Calculator [1, 2].

| **neutron cross-section parameters** | **silicon (Si)** | **aluminum (Al)** |
| --- | --- | --- |
| coherent scattering cross-section (1/cm) | 0.108 | 0.090 |
| incoherent scattering cross-section (1/cm) | 0.000 | 0.000 |
| absorption cross-section (1/cm) | 0.005 | 0.008 |

**Table S2**: Product specifications for X_T_-stage [3], Z_T_-stage [4], X_P_-stage and Y_P_-stages [5], Z_P_-stage [6], and force transducer [7].

| **component** | **parameter** | **value** |
| --- | --- | --- |
| X_T_-stage  Physik Instrumente  V-817. 096211E0 | travel range (mm) | 204 |
|  | maximum velocity (mm/s) | 3000 |
|  | resolution (nm) | 0.3 |
|  | repeatability (µm) | 2 |
|  | accuracy (µm) | 2.5 |
|  | backlash (µm) | N/A |
| Z_T_-stage  Zaber  LRQ300AL-DE51T10A | travel range (mm) | 300 |
|  | maximum velocity (mm/s) | 54 |
|  | resolution (nm) | 50 |
|  | repeatability (µm) | < 2 |
|  | accuracy (µm) | 10 |
|  | backlash (µm) | < 5 |
| X_P_-stage and Y_P_-stages  Zaber  LSQ300A-E01T3A | travel range (mm) | 300 |
|  | maximum velocity (mm/s) | 53 |
|  | resolution (nm) | 500 |
|  | repeatability (µm) | < 2 |
|  | accuracy (µm) | 90 |
|  | backlash (µm) | < 15 |
| Z_P_-stage  Newport  IDL280-Z20 | travel range (mm) | 20 |
|  | maximum velocity (mm/s) | 5 |
|  | resolution (nm) | 50 |
|  | repeatability (µm) | 0.1 |
|  | accuracy (µm) | 2 |
|  | backlash (µm) | N/A |
| force transducer  AMTI  SF3-100 | F_x_, F_y_ capacity (N) | 225 |
|  | F_z_ capacity (N) | 445 |
|  | F_x_, F_y_ sensitivity (µV/(V-N)) | 5.4 |
|  | F_z_ sensitivity (µV/(V-N)) | 1.35 |


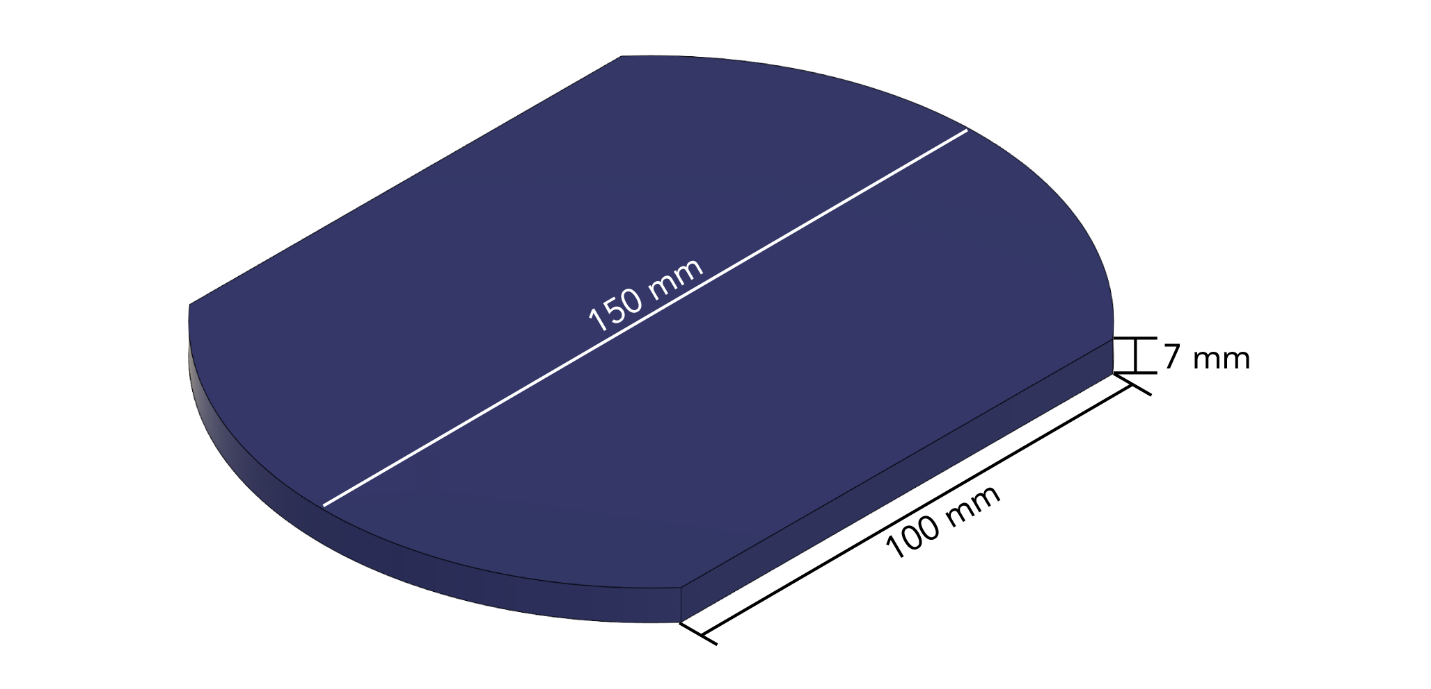


**Figure S1.** Isometric projection of custom silicon disk with relevant dimensions: thickness of 7 mm, diameter of 150 mm, flat side lengths of 100 mm.


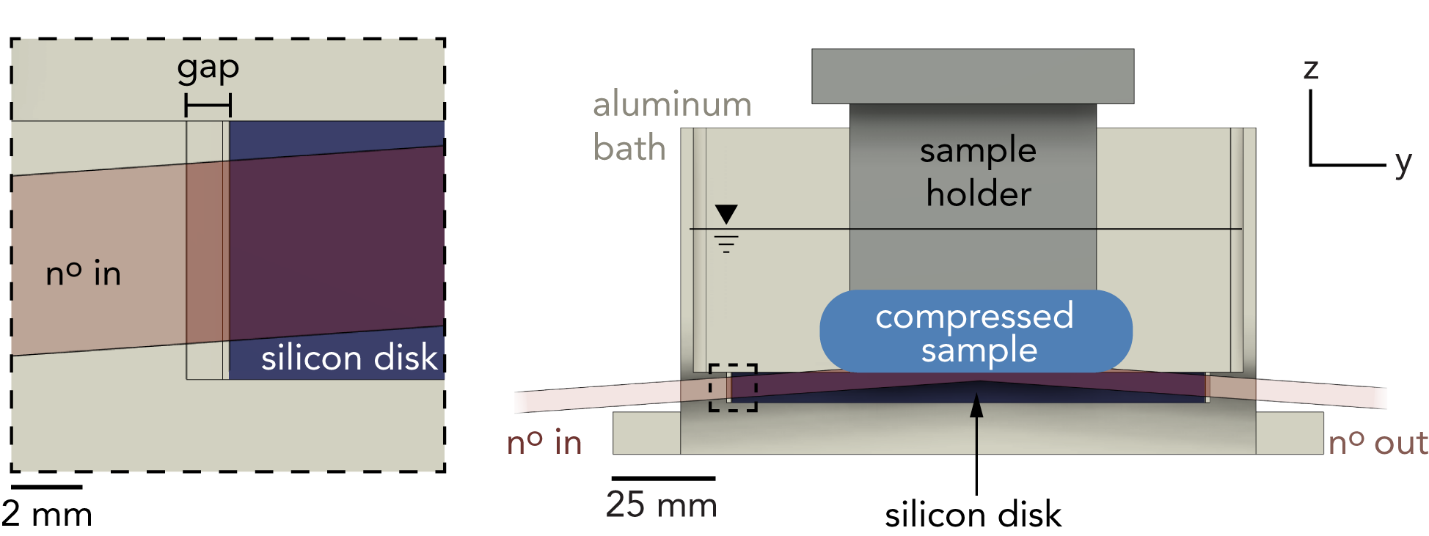


**Figure S2.** Enlarged view of the exposed interface between the aluminum bath and the silicon disk.

**
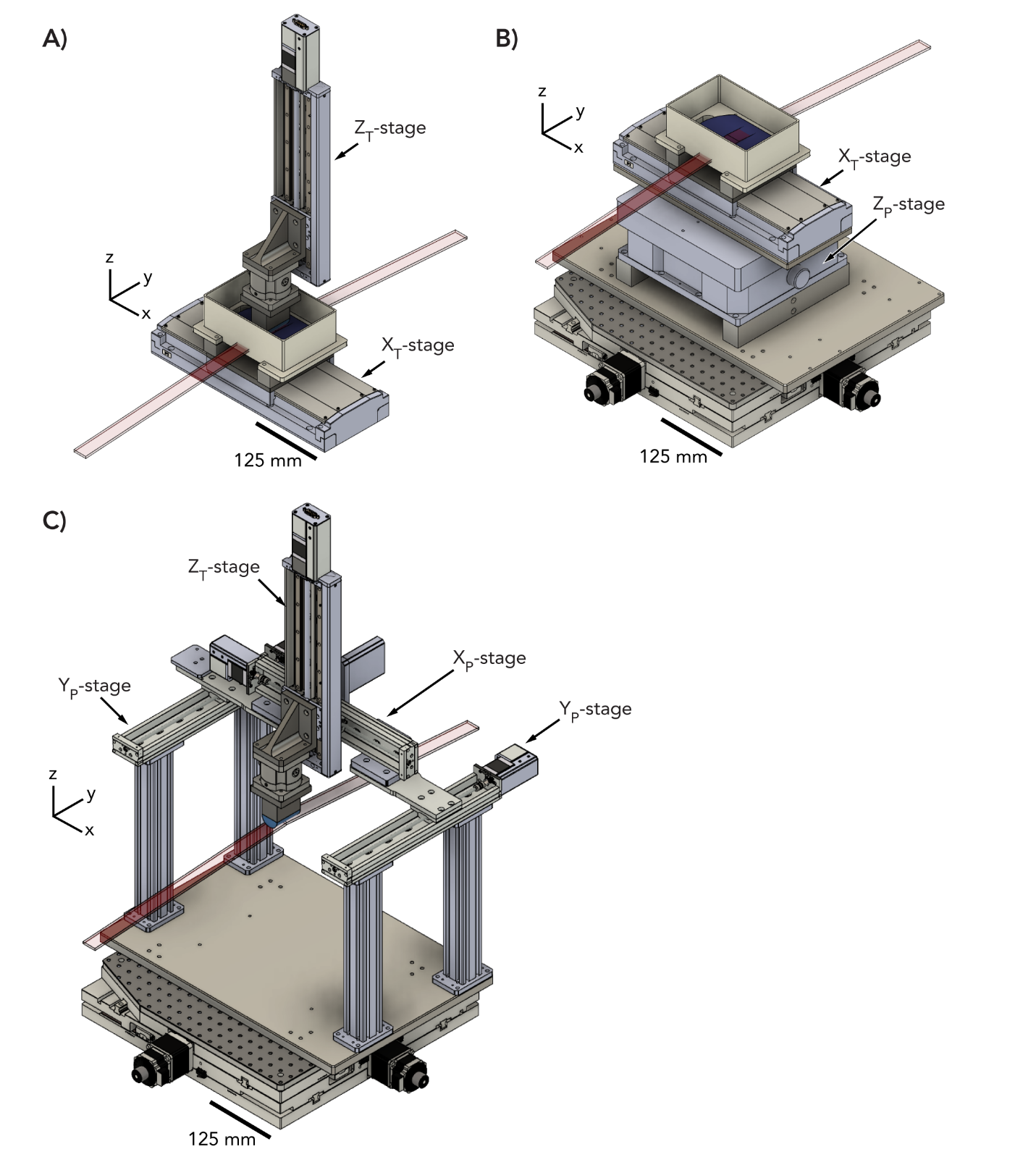
**

**Figure S3.** Isometric views of subassemblies for the **A)** tribometer, depicting the motorized vertical Z_T_ - stage and perpendicular X_T_ - stage, **B)** countersample (silicon disk) stages, showing the tribometer X_T_ - stage stacked on top of the motorized positioning Z_P_ - stage and supported by the goniometer platform below, and **C)** sample positioning X_P_ – stage and two parallel Y_P_ – stages supporting the vertical Z_T_ - stage. Red bar indicates the incident and reflected paths of neutrons going into and out of the sliding interface. The tribometer is designed such that the neutron beamline reaches the gel-silicon interface and detector without interacting with any of the motorized stages.


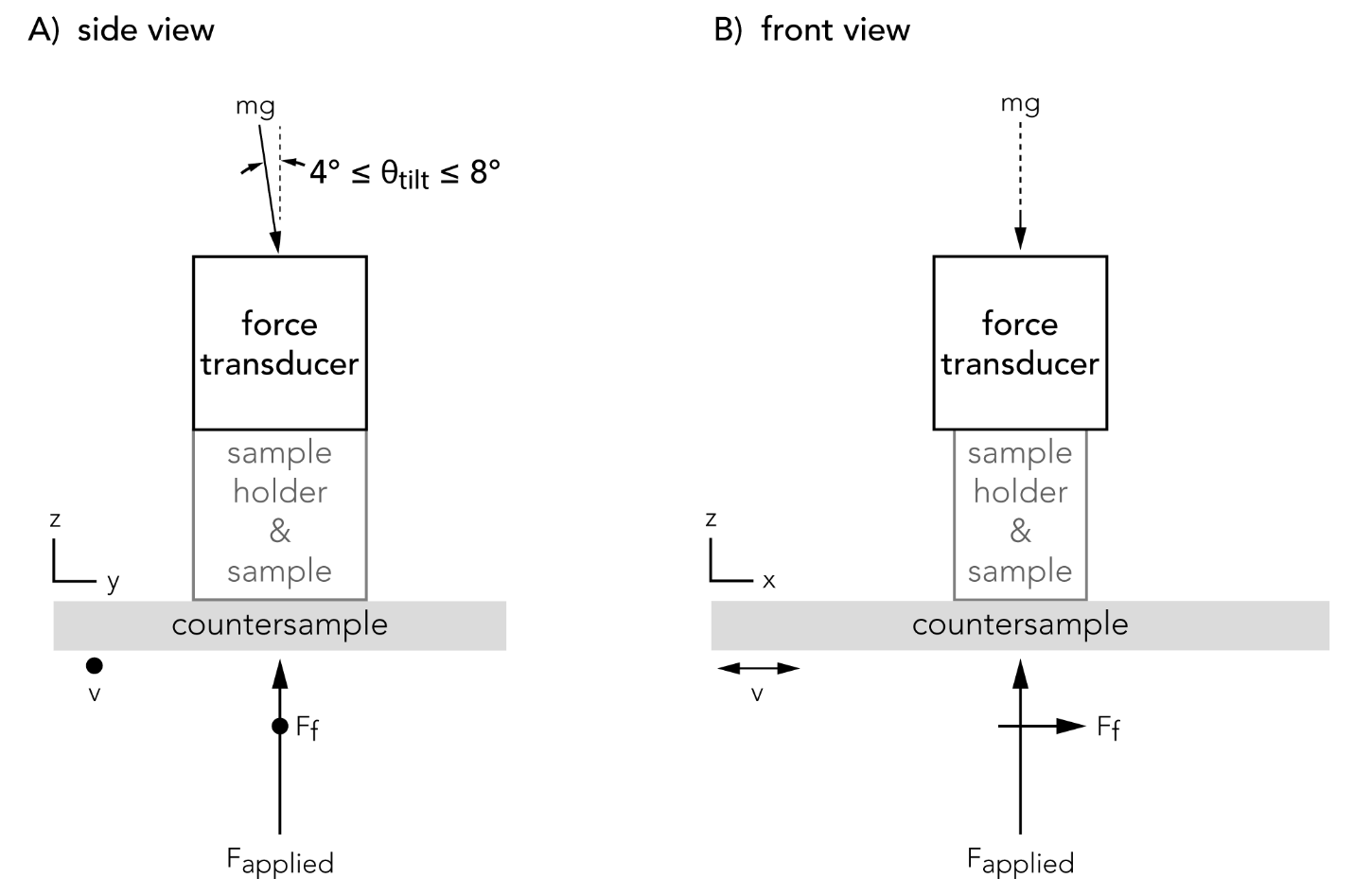


**Figure S4**. Accounting for tilt in force measurements. A) side view and B) front view schematic of the experimental configuration, including the load cell, sample holder, sample, and countersample.

**Table S3:** Fitting parameters and found values for scattering length density profiles in Figure 4C.

| **force** | **sensitivity (µV/(V-N))** | **calibration constant (mV/N)** |
| --- | --- | --- |
| F_f_ | 5.40 | 40.5763 |
| F_n_ | 1.35 | 12.3843 |


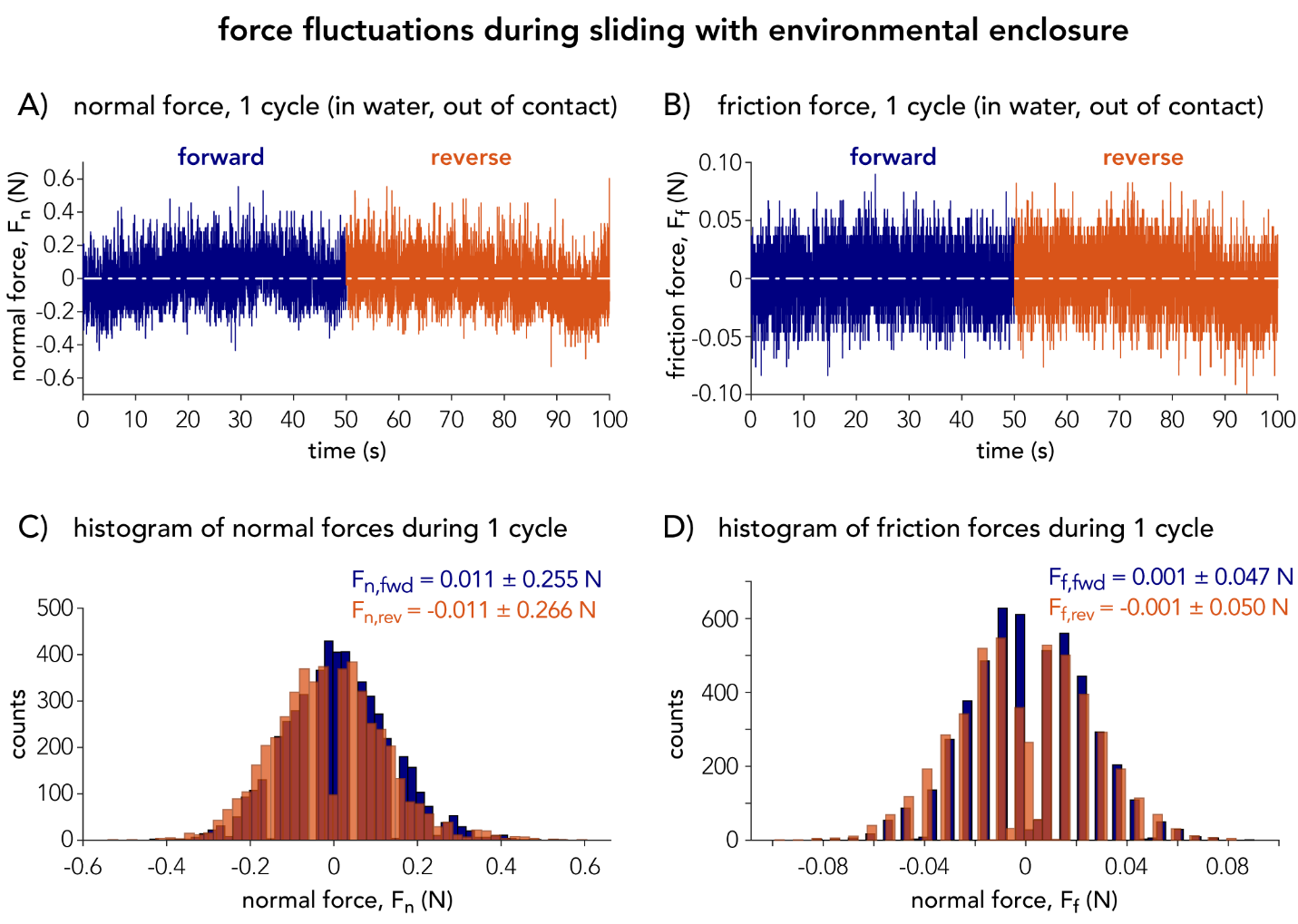


**Figure S5**. Fluctuations in (A,C) normal and (B,D) friction forces detected by the load cell due to 1 mm/s reciprocating motions with a sample holder submerged in water but out of sliding contact with an environmental enclosure.

**
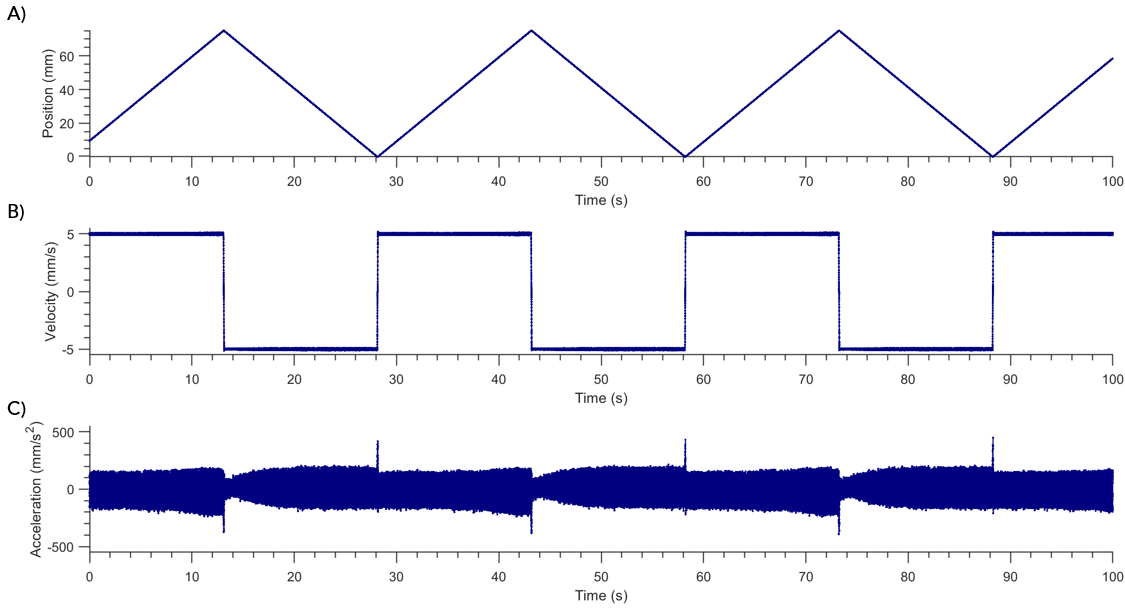
**

**Figure S6**. A) Position, B) velocity and C) acceleration profiles for the X_T_-stage motion with respect to time for multiple sliding cycles.


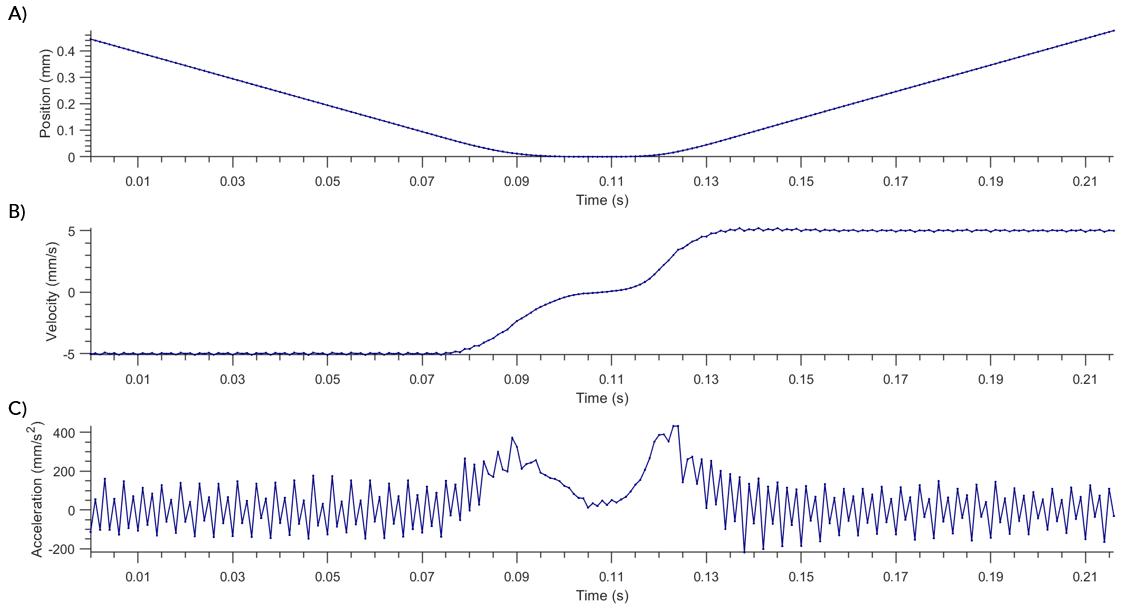


**Figure S7**. A) Position, B) velocity and C) acceleration profiles for the X_T_-stage motion with respect to time for the turnaround region of a single representative sliding cycle.


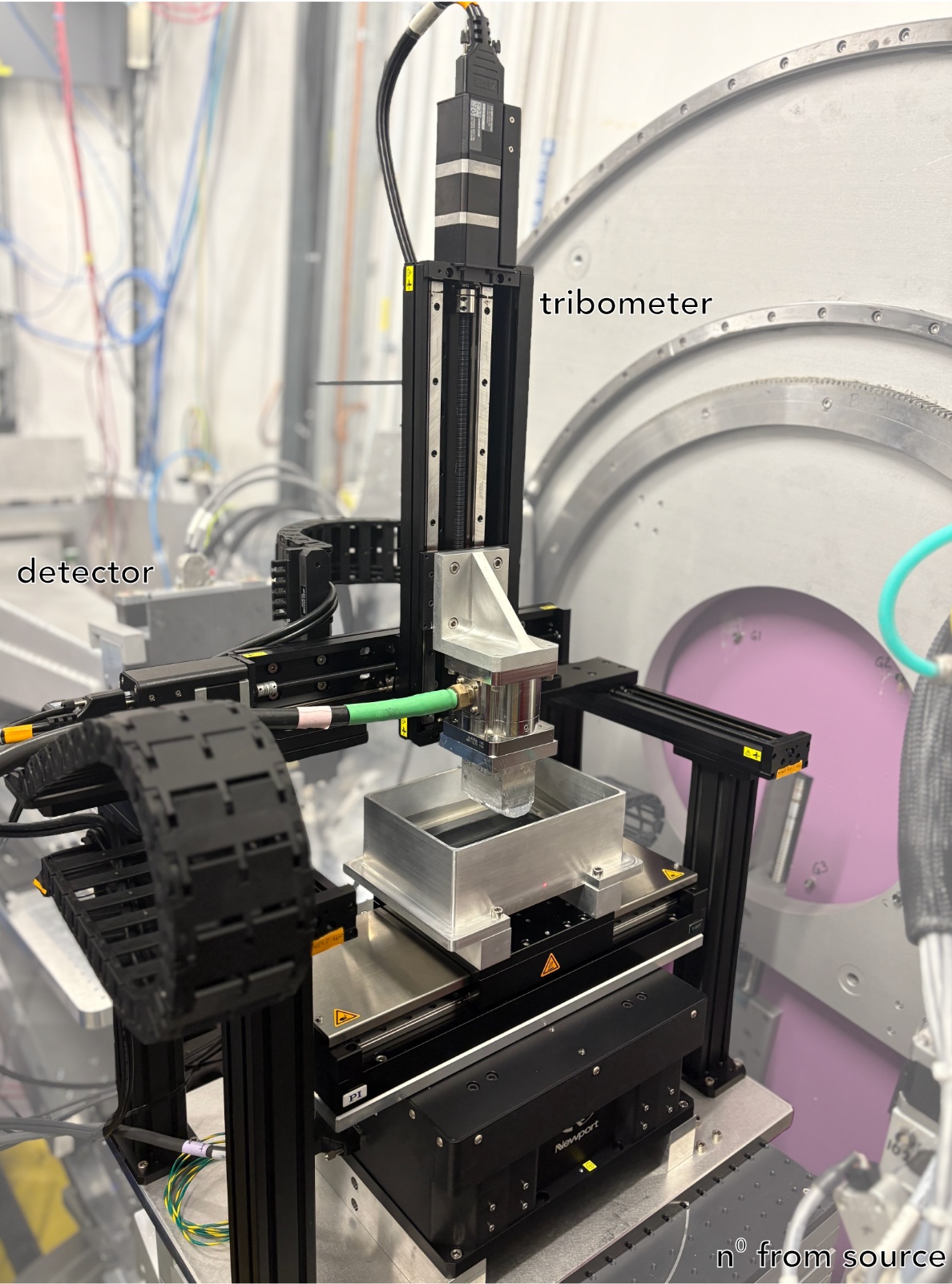


**Figure S8.** Tribometer mounted in the LIQREF at Oak Ridge National Laboratory.

**Table S4:** Fitting parameters and found values for scattering length density profiles in Figure 4C.

| **parameter** | **value** |
| --- | --- |
| position 1 background | 1.79×10^-7^ |
| position 1 intensity | 0.96 |
| position 1 D_2_O SLD | 6.27×10^-6^ Å^2^ |
| position 2 background | 1.07×10^-7^ |
| position 2 intensity | 0.97 |
| position 2 D_2_O SLD | 6.30×10^-6^ Å^2^ |
| reciprocating motion background | 1.00×10^-7^ |
| reciprocating motion intensity | 0.95 |
| reciprocating motion D_2_O SLD | 6.29×10^-6^ Å^2^ |
| Si roughness | 2.00 Å |
| SiO_2_ interface roughness | 2.00 Å |
| SiO_2_ SLD | 3.80×10^-6^ Å^2^ |
| SiO_2_ thickness | 13.04 Å |
| contaminant interface roughness | 16.40 Å |
| contaminant SLD | 5.4×10^-6^ Å^2^ |
| contaminant thickness | 33.51 Å |

**References**

1. Neutron Activation and Scattering Calculator. NIST Center for Neutron Research. https://www.ncnr.nist.gov/resources/activation/. Accessed 06 June 2025

2. Prince, E. ed: International Tables for Crystallography: Mathematical, physical and chemical tables. International Union of Crystallography, Chester, England (2006)

3. V-817 High-Load Linear Stage. PI (Physik Instrumente) L.P.. https://www.pi-usa.us/en/products/precision-motorized-linear-stages/heavy-duty-stages-for-industrial-automation/v-817-high-load-linear-stage-412418502#specification. Accessed 14 July 2025

4. LRQ300AL-DE51CT10A Specifications. Zaber Technologies Inc.. https://www.zaber.com/products/linear-stages/LRQ-DEC/specs?part=LRQ300AL-DE51CT10A. Accessed 14 July 2025

5. LSQ300A-E01T3A Specifications. Zaber Technologies Inc.. https://www.zaber.com/products/linear-stages/LSQ-E/specs?part=LSQ300A-E01T3A. Accessed 14 July 2025

6. IDL280-Z20. Newport Corporation. https://www.newport.com.cn/p/IDL280-Z20. Accessed 14 July 2025

7. SF3. Advanced Mechanical Technology, Inc.. <https://www.amti.biz/product/sf3/#specifications>. Accessed 14 July 2025
